# Increase of urinary malondialdehyde level by bisphenol A exposure: a longitudinal panel study

**DOI:** 10.1186/s12940-017-0221-9

**Published:** 2017-02-15

**Authors:** Jin Hee Kim, Yun-Chul Hong

**Affiliations:** 1 Department of Integrative Bioscience & Biotechnology, Sejong University, 209 Neungdong-ro, Gwangjin-gu, Seoul, Republic of Korea; 2 Department of Preventive Medicine, Seoul National University College of Medicine, 28 Yongon-dong, Chongno-gu, Seoul, Republic of Korea; 3 Institute of Environmental Medicine, Seoul National University Medical Research Center, 28 Yongon-dong, Chongno-gu, Seoul, Republic of Korea

**Keywords:** Elderly, Bisphenol A, Oxidative stress, Malondialdehyde

## Abstract

**Background:**

To verify oxidative stress as a possible mechanism that establishes a relationship between exposure to bisphenol A (BPA) and adverse health outcomes in the elderly Korean population, we evaluated the relation between visit-to-visit variations in urinary BPA and oxidative stress biomarker.

**Methods:**

To assess the relation between BPA and urinary malondialdehyde (MDA) as an oxidative stress biomarker, we used a mixed effect model after controlling for age, sex, BMI, drinking status, exercise, urinary cotinine level, PM_10_ on lag day 2, and mean temperature and dew point on the day. The relation between exposure to BPA and MDA level by sex of participants and polymorphisms of oxidative stress-related genes (*COX2*, *EPHX1*, *HSP70-hom*, *PON1*, *eNOS*, *CAT*, *DRD2*, *SOD2*, and *MPO*) was also evaluated.

**Results:**

A significant association was found for BPA with MDA in both male and female elderly participants (male, β = 0.19 and *p* = 0.0003; female, β = 0.18 and *p* < .0001; and total, β = 0.18 and *p* < .0001). Furthermore, the association of BPA with MDA was found regardless of any genotype of the nine oxidative stress-related genes.

**Conclusions:**

The results of our study suggest a strong association of BPA with oxidative stress, not related with sex and oxidative stress-related gene polymorphisms.

## Background

Bisphenol A (BPA) is a chemical with highest levels of production worldwide, with an annual increase of 6 to 10% [1]. The ubiquitous exposure to BPA [1] and its toxic potential [2–4] raise concerns of its adverse effects on both non-sexual and sexual organs [5–7]. Recently, several studies have suggested that oxidative stress is a possible mechanism that establishes the relation between exposure to BPA and adverse health outcomes [8, 9]. However, there has been a limited number of reports on the relation between BPA exposure and oxidative stress biomarkers [10–17], particularly for malondialdehyde (MDA) [10–13, 16]. Moreover, in previous studies, it has been difficult to capture within-subject changes because of their cross-sectional nature of the associations [10–13, 16]. For this reason, a longitudinal study with repeated measurements is required to account for within-subject changes in BPA exposure and oxidative stress levels since each subject in the panel study can be used as his or her own control with repeated measurements of rapidly changing covariates.

Therefore, in the present study, we repeatedly measured the urinary levels of BPA and MDA as a lipid peroxidation marker in the Korean elderly population, and estimated acute effect of BPA on MDA level. Furthermore, we also estimated the effect of BPA on MDA level by sex of participants and polymorphisms of oxidative stress-related genes (*COX2*, *EPHX1*, *HSP70-hom*, *PON1*, *eNOS*, *CAT*, *DRD2*, *SOD2*, and *MPO*).

## Methods

### Study population and sampling

This study estimated the relation between BPA exposure and urinary levels of MDA, an oxidative stress biomarker, in the elderly aged 60 or over recruited from the Korean Elderly Environmental Panel (KEEP) study. Briefly, among a total of 560 elderly people who visited a community elderly welfare center as many as five times for a medical examination (twice in 2008, once in 2009, and twice in 2010) [9], 548 subjects were included in the analysis after excluding 12 whose blood samples were unavailable.

### BPA measurement

We measured urinary levels of total BPA, including free and conjugated BPA, using HPLC tandem mass spectrometry (HPLC: Agilent 1200, USA; MS/MS: Agilent 6410 Triple Quad LCMS, Agilent, USA) according to previously reported procedures [9]. Shortly, five-hundred microliters of urine were buffered with 30 μL of 2.0 M sodium acetate (pH 5.0) and were then spiked with 25 μL internal standard BPA (RING-13C12, 99%; Cambridge Isotope Lab, Inc., Andover, MA, USA) and 10 μL (≥900 units) of glucuronidase/sulfatase (Sigma–Aldrich G7770, St. Louis, MO, USA). The accuracy, coefficient of precision variation, and coefficient of reproducibility variation were 99.7%, 1.0–4.7, and 0.5–5.3, respectively, based on the quality control method adopted from the Clinical and Laboratory Standards Institute (CLSI) guidelines. The limit of detection (LOD) of urinary BPA was 0.01 μg /L.

### MDA measurement

We measured urinary levels of MDA as an oxidative stress biomarker. Urinary MDA levels were determined by measuring thiobarbituric acid reactive substances [18]. Shortly, 50 μl of urine were mixed with 300 μl of 0.5 M phosphoric acid solution and 150 μl of 23 mM TBA solution (Sigma-Aldrich T-5500, Steinheim, Germany) and were heated at 95 °C for 1 h. After cooling on ice, the mixture was vortexed with 500 μl of methanol and was centrifuged at 5000 × g. The absorbance of the supernatant was measured at 532 nm using HPLC-UV with a mobile phase of potassium phosphate (0.05 mol/L; pH 6.8) and methanol (58:42, *v/v*).

### Cotinine measurement

Urinary cotinine levels were measured to monitor tobacco exposure. The cotinine level was analyzed using an enzyme-linked immunosorbent assay [18].

### Particulate matter less than 10 μm (PM_10_) concentration and meteorological factors

In a previous study for the delayed effects of PM_10_ on MDA level, significant associations of PM_10_ on lag day 2 and outdoor temperature and dew point on the day with MDA level were found [18] and thus we adjusted for these factors in our models. Data was acquired from the Korea National Institute of Environmental Research for PM_10_ on lag day 2 at the monitoring center nearest to the residence of each participant [18]. The outdoor temperature and dew point measured at the Songwol-dong monitoring center nearest to the residence of the study participants during the study period were obtained from the Korea Meteorological Administration [18].

### Genotyping of oxidative stress-related genes

Genomic DNA was extracted from peripheral blood lymphocytes using a QIAamp DNA Blood Mini Kit (Qiagen, Valencia, CA, USA), and twenty-one polymorphisms of nine oxidative stress-related genes *–* cyclooxygenase 2 (*COX2*), epoxidehydrolase 1 (*EPHX1*), heat shock protein 70-hom (*HSP70-hom*), paraoxonase 1 (*PON1*), endothelial nitric oxide synthase (*eNOS*), catalase (*CAT*), dopamine receptor D2 (*DRD2*), superoxide dismutase 2 (*SOD2*), and myeloperoxidase (*MPO*) – were determined using the TaqMan fluorogenic 5′ nuclease assay (rs5277 for *COX2*, rs3766934, rs1051740, and rs2234922 for *EPHX1*, rs2227956 and rs2075800 for *HSP70-hom*, rs854560, rs13306698, and rs662 for *PON1*, rs1799983 for *eNOS*, rs769218 and rs769217 for *CAT*, rs1800497 for *DRD2*, rs4880, rs2758331, and rs5746136 for *SOD2*, and rs7208693 for *MPO*) and a single base primer extension assay (rs3218625 for *COX2*, rs2853796 and rs7830 for *eNOS*, and rs2071409 for *MPO*). Negative controls were included to ensure genotyping accuracy. For confirmation, five percent of the samples were randomly chosen and genotyped again, producing identical results.

For the TaqMan fluorogenic 5′ nuclease assay (ABI, Foster City, CA, USA), the final volume of polymerase chain reaction (PCR) was 5 μl, containing 10 ng of genomic DNA and 2.5 μl TaqMan Universal PCR Master Mix, with 0.25 μl of 20X or 0.125 μl of 40X Assay Mix (Assay ID, AHVI68H for rs5277, C___2725995_20 for rs3766934, C_____14938_30 for rs1051740, C__11638783_30 for rs2234922, C__25630755_10 for rs2227956, C___3052613_1_ for rs2075800, AHT9819 for rs854560, C__31373257_10 for rs13306698, C___2548962_20 for rs662, C___3219460_20 for rs1799983, C___3102900_10 for rs769218, C___3102907_10 for rs769217, C___7486676_10 for rs1800497, C___8709053_10 for rs4880, C__16288770_10 for rs2758331, C__29322854_10 for rs5746136, and C__25609936_10 for rs7208693). All polymerase chain reactions and endpoint fluorescent readings were conducted according to previously reported procedures [9]. For the single base primer extension assay, SNaPShot assay kit (ABI, Foster City, CA, USA) was used according to previously reported procedures [9].

The primers and probes designed for rs5277, rs3218625, rs854560, rs2853796, rs7830, and rs2071409 were as follows:

- rs5277-forward, 5′-TCCCTTCCTTCGAAATGCAATTATGA-3′,
- rs5277-reverse, 5′-GCTAAAAACCTTAGAAAGACACTTGT-3′,
- rs5277-VIC, 5′-CTTACATGTCAACACATAAC-3′,
- rs5277-FAM, 5′-ACATGTCAAGACATAAC-3′,
- rs3218625-forward, 5′-ATTCAGTGTTCCAGATCCAGAG-3′,
- rs3218625-reverse, 5′-AAATAAATATGATCATTAGACTTCTACAGTTC-3′,
- rs3218625-SNP, 5′-CATCAATGCAAGTTCTTCCCGMTCC-3′,
- rs854560-forward, 5′-ACAACCTGTACTTTCTGTTCTCTTTTCTG-3′,
- rs854560-reverse, 5′-GAAAACACTCACAGAGCTAATGAAAGC-3′,
- rs854560-VIC, 5′-CAGTATCTCCAAGTCTTC-3′,
- rs854560-FAM, 5′-CAGTATCTCCATGTCTTC-3′,
- rs2853796-forward, 5′-TTCCTGTSCCAGAGGCAG-3′,
- rs2853796-reverse, 5′-GACAAGGTTGTCACAGGGC-3′,
- rs2853796-SNP, 5′- CCYTGAAGCCGTCCCTGGGGCTGGG-3′,
- rs7830-forward, 5′- ATTCTGGCAGGAGCGGCT-3′,
- rs7830-reverse, 5′-TCTGTCCCTAGATTGTGTGACTC-3′,
- rs7830-SNP, 5′-ACTCCCTTCAGGCAGTCCTTTAGTC-3′,
- rs2071409-forward, 5′- TGCCAGCCCAGAATATCC-3′,
- rs2071409-reverse, 5′-GCTGCATGCTGAACACAC-3′,
- rs2071409-SNP, 5′-CACAGTGTCCATGGGTGTTCCCC-3′.

The probes for rs1051740, rs2234922, rs13306698, and rs662 were DME, and those for rs3766934, rs2227956, rs2075800, rs1799983, rs769218, rs769217, rs1800497, rs4880, rs2758331, rs5746136, and rs7208693 were pre-designed.

### Statistical analysis

The BPA concentrations under the LOD were assigned as a default value of LOD concentration divided by 2. Since the detection range for cotinine was 1–10,000 mg/L, the cotinine level was assigned as 0.5 mg/L for values less than 1 mg/L and 15,000 mg/L for values greater than 10,000 mg/L. Because the present panel study conducted repeated measurements of urinary BPA and MDA at several time points for each individual (five measurements at maximum for both exposure and outcome), we used a mixed effect model with repeated values of BPA and MDA levels to assess the relation of visit-to-visit variations in BPA exposure with MDA levels in order to evaluate the short-term effects of the changes in BPA exposure levels over time. In the model, we adjusted for age, sex, body mass index (BMI), drinking status, exercise, urinary cotinine level, PM_10_ on lag day 2, and mean temperature and dew point on the day because these factors affected the MDA level significantly. Age, BMI (weight (kg)/ height^2^ (m^2^)), cotinine levels, PM_10_ on lag day 2, and mean temperature and dew point on the day were treated as continuous variables, and sex, drinking status, and exercise were treated as categorical variables in the models. We also estimated the relation between BPA and MDA levels by sex and by the genetic polymorphisms of *COX2*, *EPHX1*, *HSP70-hom*, *PON1*, *eNOS*, *CAT*, *DRD2*, *SOD2*, and *MPO*. Furthermore, we calculated intra-class correlation coefficients (ICCs) - defined as the ratio of inter-individual variance to total variance - of BPA and MDA to evaluate the intra- and inter-individual variations of repeated BPA and MDA measures. SAS version 9.3 (SAS Institute Inc., Cary, NC, USA) was used for statistical analyses with a significance level of *p* < 0.05.

## Results

The participants in our study were a total of 548 elderly people, 142 males and 406 females (Table 1). At baseline, the mean age of the participants was 70.8 years, and the number of obese participants with BMI ≥ 25 was 242 (44.2%). Current smokers, drinkers, and exercisers were 5.5, 22.1, and 61.5%, respectively, and male participants smoked and consumed alcohol more than female participants (both *p* < .0001). The mean number of visits of the participants was 3.3, and females participated more actively compared to males (*p* = 0.0847).

**Table 1:** Demographic characteristics of the participants

Characteristic	Total	Male	Female	*p*-Value
No. of participants (%)	548 (100)	142 (25.9)	406 (74.1)	
Visit number [mean ± SE]	3.3 ± 0.1	3.2 ± 0.1	3.4 ± 0.1	0.0847
Mean age (min-max), year	70.8 (60–87)	71.4 (62–84)	70.5 (60–87)	0.0653
Height [mean ± SE (cm)]	154.7 ± 0.3	164.3 ± 0.4	151.3 ± 0.3	<.0001
Weight [mean ± SE (Kg)]	59.4 ± 0.4	65.8 ± 0.8	57.1 ± 0.4	<.0001
BMI (kg/m^2^), no. (%)				
≥ 25	242 (44.2)	56 (39.4)	186 (45.8)	0.1485
23 ~ <25	169 (30.8)	42 (29.6)	127 (31.3)	
< 23	137 (25.0)	44 (31.0)	93 (22.9)	
No. of current smokers (%)	30 (5.5)	29 (20.4)	1 (0.2)	<.0001
No. of drinker (%)	121 (22.1)	78 (54.9)	43 (10.6)	<.0001
Exercise, no. of yes (%)	337 (61.5)	88 (62.0)	249 (61.3)	0.9260

BPA, MDA, and cotinine were measured in a total of 1625, 1637, and 1632 urine samples, respectively (Table 2). The mean levels (inter-quartile ranges) of urinary BPA, MDA, and cotinine were 1.2 μg/L (0.4–1.2 μg /L), 1.9 μmol/L (1.1–2.4 μmol/L), and 274.7 mg/L (0.5–4.5 mg/L), respectively. In particular, 95 percentile and maximum levels of urinary BPA were 3.7 μg/L and 67.6 μg/L, respectively, and number of urine samples with BPA concentrations under the LOD was 32. In the evaluation for intra- and inter-individual variations of BPA and MDA levels, ICC of BPA was 0.11 and that of MDA was 0.07. The means for PM_10_ on lag day 2 of the health examination and temperature and dew point on the day were 41.3 μg/m^3^, 16.8 °C, and 6.0 °C, respectively.

**Table 2:** Distribution of repeated BPA, MDA, cotinine, PM_10_, temperature, and dew point

			Selected percentiles		
Chemicals	n	Mean (SD)	25th	50th	75th
BPA (μg/L)	1625	1.2 (2.6)	0.4	0.7	1.2
MDA (μmol/L)	1637	1.9 (1.2)	1.1	1.7	2.4
Cotinine (mg/L)	1632	274.7 (1564.1)	0.5	2.1	4.5
PM_10_ on lag day 2 (μg/m^3^)	1762	41.3 (23.6)	26.4	36.4	52.5
Temperature on the day (°C)	1818	16.8 (9.0)	9.8	18.0	24.9
Dew point on the day (°C)	1818	6.0 (10.8)	−2.0	7.7	15.3

Twenty-one genotyped polymorphisms of *COX2*, *EPHX1*, *HSP70-hom*, *PON1*, *eNOS*, *CAT*, *DRD2*, *SOD2*, and *MPO* are listed in Table 3. The call rate of twenty polymorphisms, except rs662, was high with a minimum of 98.7% (93.5% for rs662), and all replicated genotyping showed identical results with an accuracy of 100% (Table 3). When we tested for the Hardy-Weinberg equilibrium (HWE) of each polymorphism with genotype frequency, the study participants were in HWE for twenty polymorphisms, except rs2227956 (*p* < 0.05 for rs2227956 and *p* > 0.05 for the other twenty polymorphisms using a *χ* ^2^ test).

**Table 3:** Genotyped polymorphisms

Gene	rs no.	HGVS name	Chromosome no.	Position	Amino acid change	Call rate (%)	Accuracy (%)
*COX2*	rs5277	c.306G > C	1	Codon102	Val102=	99.8	100
	rs3218625	c.1759G > A	1	Codon587	Gly587Arg	100	100
*EPHX1*	rs3766934	c.-5-1409G > T	1	Intron	-	99.4	100
	rs1051740	c.337 T > C	1	Codon113	Tyr113His	99.6	100
	rs2234922	c.416A > G	1	Codon139	His139Arg	98.7	100
*HSP70-hom*	rs2227956	c.1478C > T	6	Codon493	Met493Thr	99.1	100
	rs2075800	c.1804G > A	6	Codon602	Glu602Lys	99.5	100
*PON1*	rs854560	c.163 T > A	7	Codon55	Leu55Met	99.5	100
	rs13306698	c.478A > G	7	Codon160	Arg160Gly	99.8	100
	rs662	c.575A > G	7	Codon192	Gln192Arg	93.5	100
*eNOS*	rs1799983	c.894 T > G,	7	Codon298	Asp298Glu	99.5	100
	rs2853796	c.1821-62G > T	7	Intron	-	99.5	100
	rs7830	c.3106 + 11G > T	7	Intron	-	99.5	100
*CAT*	rs769218	c.67-60G > A	11	Intron	-	100	100
	rs769217	c.1167C > T	11	Codon389	Asp389=	98.7	100
*DRD2*	rs1800497	c.2137G > A	11	Codon713	Glu713Lys	99.6	100
*SOD2*	rs4880	c.47 T > C	16	Codon16	Val16Ala	99.2	100
	rs2758331	c.523 + 816G > T	16	Intron	-	99.6	100
	rs5746136	c.*441G > A	16	Downstream	-	99.8	100
*MPO*	rs7208693	c.157G > T	17	Codon53	Val53Phe	99.3	100
	rs2071409	c.2031-6A > C	17	Intron	-	99.5	100

The evaluation of the relation between BPA and MDA levels indicated a strong association for BPA exposure with an increase in MDA level (β = 0.18, 95% confidence interval (CI): 0.14, 0.23, and *p* < .0001) regardless of sex (male, β = 0.19, 95% CI: 0.09, 0.29, and *p* = 0.0003; and female, β = 0.18, 95% CI: 0.12, 0.23, and *p* < .0001) (Table 4). To evaluate the relation of BPA with MDA according to the genotype of oxidative stress-related genes, the relation between BPA and MDA was estimated for each genetic polymorphism and was found to be consistent regardless of any genotype of *COX2*, *EPHX1*, *HSP70-hom*, *PON1*, *eNOS*, *CAT*, *DRD2*, *SOD2*, and *MPO* (Table 4). Furthermore, we explored the pattern of dose–response relationship between BPA and MDA levels, but did not find any trend for non-linear relationship between the two.

**Table 4:** The relation of BPA with oxidative stress by genotypes of oxidative stress-related genes

			N	Observation	β	Lower 95% CI	Upper 95% CI	*p*-Value
Total			517	1528	0.18	0.14	0.23	<.0001
Male			134	365	0.19	0.09	0.29	0.0003
Female			383	1163	0.18	0.12	0.23	<.0001
*COX2*	rs5277	GG	468	1392	0.17	0.12	0.22	<.0001
		GC	47	131	0.26	0.13	0.38	0.0001
		CC	1	3	-	-	-	-
		GC + CC	48	134	0.26	0.13	0.38	0.0001
	rs3218625	GG	500	1480	0.18	0.14	0.23	<.0001
		GA	17	48	0.19	−0.04	0.42	0.1031
		AA	0	0	-	-	-	-
		GA + AA	17	48	0.19	−0.04	0.42	0.1031
*EPHX1*	rs3766934	GG	329	996	0.17	0.12	0.23	<.0001
		GT	164	472	0.20	0.11	0.29	<.0001
		TT	22	53	0.22	−0.02	0.46	0.0667
		GT + TT	186	525	0.20	0.12	0.28	<.0001
	rs1051740	TT	172	501	0.16	0.08	0.24	0.0001
		TC	258	768	0.19	0.12	0.25	<.0001
		CC	83	250	0.22	0.10	0.33	0.0003
	rs2234922	AA	380	1129	0.18	0.13	0.24	<.0001
		AG	125	365	0.20	0.12	0.29	<.0001
		GG	4	12	0.93	−3.41	5.27	0.4539
		AG + GG	129	377	0.20	0.12	0.29	<.0001
*HSP70-hom*	rs2227956	TT	408	1202	0.18	0.13	0.23	<.0001
		TC	103	310	0.18	0.05	0.31	0.0084
		CC	1	2	-	-	-	-
		TC + CC	104	312	0.18	0.05	0.31	0.0061
	rs2075800	GG	181	541	0.20	0.12	0.28	<.0001
		GA	242	718	0.14	0.07	0.21	<.0001
		AA	91	261	0.22	0.12	0.33	<.0001
*PON1*	rs854560	TT	455	1342	0.19	0.14	0.23	<.0001
		TA	57	174	0.13	−0.02	0.28	0.0783
		AA	2	4	-	-	-	-
		TA + AA	59	178	0.14	−0.01	0.29	0.0669
	rs13306698	AA	451	1328	0.19	0.15	0.24	<.0001
		AG	62	187	0.17	−0.01	0.35	0.0687
		GG	3	9	-	-	-	-
		AG + GG	65	196	0.16	−0.01	0.34	0.0662
	rs662	GG	211	636	0.16	0.09	0.23	<.0001
		GA	209	597	0.20	0.11	0.28	<.0001
		AA	64	193	0.24	0.13	0.36	<.0001
*eNOS*	rs1799983	GG	441	1309	0.18	0.13	0.23	<.0001
		GT	71	206	0.15	0.01	0.28	0.0308
		TT	2	7	-	-	-	-
		GT + TT	73	213	0.15	0.01	0.28	0.0299
	rs2853796	TT	202	589	0.20	0.13	0.27	<.0001
		TG	241	720	0.17	0.10	0.24	<.0001
		GG	71	210	0.15	0.02	0.28	0.0247
	rs7830	GG	158	453	0.23	0.14	0.31	<.0001
		GT	252	741	0.17	0.10	0.25	<.0001
		TT	105	327	0.16	0.08	0.25	0.0001
*CAT*	rs769218	GG	171	484	0.14	0.05	0.22	0.0016
		GA	259	787	0.19	0.13	0.25	<.0001
		AA	86	256	0.26	0.13	0.38	<.0001
	rs769217	CC	172	485	0.14	0.05	0.22	0.0016
		CT	254	772	0.19	0.13	0.26	<.0001
		TT	82	246	0.25	0.12	0.38	0.0002
*DRD2*	rs1800497	GG	197	583	0.19	0.11	0.26	<.0001
		GA	232	679	0.20	0.13	0.26	<.0001
		AA	86	259	0.15	0.02	0.28	0.0276
*SOD2*	rs4880	TT	400	1189	0.20	0.14	0.25	<.0001
		TC	106	306	0.12	0.00	0.23	0.0430
		CC	6	20	0.66	−0.04	1.35	0.0624
		TC + CC	112	326	0.15	0.04	0.26	0.0074
	rs2758331	GG	406	1204	0.20	0.14	0.25	<.0001
		GT	103	299	0.12	0.00	0.23	0.0500
		TT	5	17	0.64	−0.29	1.56	0.1438
		GT + TT	108	316	0.15	0.04	0.26	0.0094
	rs5746136	GG	167	520	0.17	0.09	0.26	<.0001
		GA	260	750	0.16	0.09	0.22	<.0001
		AA	88	255	0.25	0.14	0.35	<.0001
*MPO*	rs7208693	GG	408	1199	0.19	0.14	0.24	<.0001
		GT	97	293	0.16	0.03	0.29	0.0197
		TT	8	24	-	-	-	-
		GT + TT	105	317	0.15	0.02	0.27	0.0224
	rs2071409	AA	442	1300	0.19	0.13	0.24	<.0001
		AC	70	214	0.17	0.06	0.27	0.0016
		CC	2	5	-	-	-	-
		AC + CC	72	219	0.17	0.07	0.27	0.0011

Adjusted for age, sex, BMI, drinking status, exercise, urinary cotinine level, PM_10_ on lag day 2, and mean temperature and dew point on the day

## Discussion

This study showed a strong association of BPA with MDA, not related with sex or with the genetic polymorphisms of nine oxidative stress-related genes (*COX2*, *EPHX1*, *HSP70-hom*, *PON1*, *eNOS*, *CAT*, *DRD2*, *SOD2*, and *MPO*).

Previous reports on the relation between BPA exposure and oxidative stress have supported the possibility of BPA exposure having an effect on adverse health outcomes through oxidative stress. Previous research reported the in vitro induction of reactive oxygen species by BPA in mouse Neuro2a and GC1 cells [14] and a positive correlation of BPA exposure with urinary level of DNA oxidation marker, 8-hydoxydeoxyguanosine (8-OHdG), in residents living in and around e-waste dismantling facilities of China [17]. Furthermore, a longitudinal panel study for pregnant women found positive associations of BPA exposure with urinary oxidative stress markers, 8-OHdG and isoprostane [15]. However, evidence of the relation between BPA exposure and MDA level was inconclusive. Animal studies for BPA observed an increase in MDA level in the heart, liver, ovary, and renal tissues of Wistar albino rats that had been orally administered a high dose of BPA (10 mg/kg/day or 25 mg/kg/day for durations between 30 days and 60 days) [10, 11, 13]. An increase in oxidative stress biomarkers due to BPA exposure was observed in several epidemiologic studies as well. Oxidative stress markers, such as 8-OHdG, white blood cell count, and C-reactive protein, as well as MDA increased in postmenopausal women exposed to BPA, even though the phenomenon was not shown in men and in premenopausal women [16]. However, in a cross-sectional study for adults, BPA was not associated with MDA and 8-OHdG levels after adjusting for covariates affecting oxidative stress [12]. Although BPA was found to affect oxidative stress levels in animals and in older females, a longitudinal panel study found no evidence of a change in lipid peroxidation by BPA exposure. Therefore, the present study estimated the effect of real-time BPA exposure on MDA level, and the results indicate a statistically significant increase in the MDA level related to the BPA exposure, indicating that exposure to BPA at low levels in the environment might be able to cause oxidative damage in elderly individuals, resulting in the development of oxidative stress-related diseases.

In the present study, we tried to estimate the difference of the effect that BPA exposure had on MDA level by sex and genetic polymorphisms of oxidative stress-related genes (*COX2*, *EPHX1*, *HSP70-hom*, *PON1*, *eNOS*, *CAT*, *DRD2*, *SOD2*, and *MPO*) because the effect of BPA exposure on adverse health outcomes related to oxidative stress was found to be different depending on sex or genetic polymorphisms of oxidative stress-related genes [9, 16]. However, we did not find any difference on the effect of BPA exposure on MDA level by sex and by the genetic polymorphisms stated above. BPA is a non-persistent chemical with a short biological half-life < 6 h, and the oxidative stress that increases due to BPA exposure might be quickly repaired by defense systems in the body [14]. We evaluated the short-term effects of the changes in BPA exposure on MDA level and found no difference on the effect of BPA exposure on MDA level by sex and the tested genetic polymorphisms, which may be due to a momentary effect of BPA on the MDA level made before the defense system of the body becomes active. However, humans are ubiquitously exposed to BPA, and chronic exposure might have an effect on various adverse health outcomes through the continuous accumulation of oxidative stress. Therefore, improving antioxidant defenses, such as with antioxidant supplementation, and regulating BPA exposure in the elderly population could potentially prevent oxidative stress resulting in oxidative stress-related diseases.

In the present study, ICC of BPA was 0.11 while that of MDA was 0.07, indicating that MDA was more changeable than BPA for each individual even though intra-individual variation was larger than inter-individual variation for both BPA and MDA. It is explainable based on several points. First, although temporal BPA exposure levels in the same individual were correlated each other in our study because lifestyle habit of each individual is not changed a bit, intra-individual variation of BPA exposure can be still high because half-life of BPA is less than 6 h and participants may be exposed to BPA through various exposure sources every day. Second, given that MDA is a nonspecific proxy variable, the covariates controlled in our model, such as age, sex, BMI, drinking status, exercise, urinary cotinine level, PM_10_ on lag day 2, and mean temperature and dew point on the day, can easily affect MDA level. In fact, all these factors significantly affected MDA level in our analysis. For this reason, we adjusted for these covariates affecting MDA level in the model and found a strong and consistent association of BPA level with MDA level even after adjustment for these covariates.

The major sources of human exposures to BPA are thought to be food and beverage consumption, because BPA is employed to make polycarbonate plastics and epoxy resins used in a variety of common consumer products including water pipes and beverage cans [19–21]. A recent study reported that urinary BPA concentrations increased more than 1000% in subjects who consumed one can of soup per day for 5 days compared to subjects who ate fresh soup [22]. These results indicated that BPA leaches out of source materials in normal condition of use, which can be accelerated if the materials are exposed to high temperatures or acidic environments [23, 24]. Although data on daily BPA intake was not available in the present study, the previous study showing a significant increase of BPA by canned food consumption supports a possibility that food and beverage consumption may be major sources of BPA exposure in Korean elderly frequently consuming canned food.

The strengths of the present study merit further discussion. First, to the best of our knowledge, this is the first longitudinal panel study to investigate the effect of BPA exposure on MDA levels with repeated measurements for BPA and MDA levels for each participant. The design of this panel study allows for the evaluation of the short-term effects on MDA by temporal BPA exposure level. Moreover, this longitudinal study served the subjects as their own controls over the study period. Since our study purpose was to evaluate the acute effect of BPA, a non-persistent chemical with a biological half-life < 6 h on the MDA level, we used a mixed effect model to evaluate the short-term effects of the changes in BPA exposure levels on MDA level. However, the effect of chronic BPA exposure on oxidative stress should be further studied in the future.

Our study had limitations as well. We recruited subjects aged 60 years or older. If age modifies the effect of BPA on the MDA level, our results may not be generalized to a younger population. In addition, we did not consider other environmental exposure that the participants may be co-exposed to during the present study, affecting MDA levels, even though PM_10_ and meteorological factors were controlled in the models. Since other forms of environmental exposure could also be associated with oxidative stress, the combined effect of multiple exposure factors inducing oxidative stress should be further studied.

## Conclusions

Overall, short-term exposure to BPA was significantly associated with MDA, an oxidative stress biomarker in the elderly. The association between BPA exposure and MDA level was found regardless of sex and any genotype of nine tested oxidative stress-related genes, indicating the strong association of BPA with MDA levels. These findings shed new light to understand physiological mechanism on the development of a variety of diseases by BPA.

## Abbreviations

8-OHdG: 8-Hydoxydeoxyguanosine
BMI: Body mass index
BPA: Bisphenol A
*CAT*: Catalase
CI: Confidence interval
*COX2*: Cyclooxygenase 2
*DRD2*: Dopamine receptor D2
*eNOS*: Endothelial nitric oxide synthase
*EPHX1*: Epoxidehydrolase 1
*HSP70-hom*: Heat shock protein 70-hom
HWE: Hardy-Weinberg equilibrium
ICC: Intra-class correlation coefficient
LOD: Limit of detection
MDA: Malondialdehyde
*MPO*: Myeloperoxidase
PM_10_: Particulate matter less than 10 μm
*PON1*: Paraoxonase 1
*SOD2*: Superoxide dismutase 2
